# Antioxidant and Antibacterial Activities of Chinese Native Thyme Essential Oils with Different Chemotypes

**DOI:** 10.3390/molecules29246035

**Published:** 2024-12-21

**Authors:** Meiyu Sun, Yanan Zhang, Yuanpeng Hao, Jiahui Miao, Guofeng Sun, Jianhua Xiao, Xiao Yang, Jinzheng Zhang, Lei Shi

**Affiliations:** 1 State Key Laboratory of Plant Diversity and Specialty Crops, Institute of Botany, Chinese Academy of Sciences, Beijing 100093, China; sunmeiyu@ibcas.ac.cn (M.S.); zhangyanan@ibcas.ac.cn (Y.Z.); yphao@ibcas.ac.cn (Y.H.); miaojiahui@ibcas.ac.cn (J.M.); yangxiao22@mails.ucas.ac.cn (X.Y.); 2 China National Botanical Garden, Beijing 100093, China; sungf@ibcas.ac.cn (G.S.); xiaojianhua@ibcas.ac.cn (J.X.); 3 University of Chinese Academy of Sciences, Beijing 100049, China

**Keywords:** Chinese native thyme, essential oil, chemotype, antioxidant activity, antibacterial activity

## Abstract

Thyme essential oils (EOs) have antioxidant, antiviral, antifungal, antibacterial, anti-inflammatory, and immunological properties and are used in medicine, food, feed additives, and cosmetics. Here, we made use of a multidimensional analytical method to analyze the differences in the chemical components, chemotypes, and antioxidant and antibacterial activities of EOs from 24 Chinese native thymes. These Chinese native thymes comprised 10 species (*Thymus quinquecostatus*, *T. mongolicus*, *T. inaequalis*, *T. mandschuricus*, *T. curtus*, *T. amurensis*, *T. roseus*, *T. proximu*, *T. marschallianus*, and *T. altaicus*) and two varieties (*T. quinquecostatus* var. *asiaticus* and *T. quinquecostatus* var. *przewalskii*). Four primary chemotype groups were identified, namely carvacrol, thymol, geraniol, and α-terpineol. The maximum carvacrol, thymol, geraniol, and α-terpineol contents were 72.4, 58.6, 59.5, and 65.4%, respectively. The antioxidant capacities of the thymol and carvacrol chemotype EOs were found to be significantly superior to the other chemotypes using three antioxidant assays: DPPH, ABTS, and FRAP. Moreover, the thymol and carvacrol EO chemotypes could significantly inhibit the growths of the common food-borne pathogenic bacteria *Staphylococcus aureus* and *Escherichia coli*. A correlation analysis between the EO components and the bacteria showed that thymol significantly positively correlated with the bacteria. In summary, we analyzed the thyme EOs’ antioxidant and antibacterial activities, which laid a foundation for their use in medicines, foods, feed additives, and cosmetics. The results will also be very useful for the selection of wild thymes for functional research on carvacrol-, thymol-, geraniol-, and α-terpineol-rich essential oil chemotypes and the product development of feed additives, cosmetics, etc.

## 1. Introduction

Thyme (*Thymus* genus) is a medicinal and aromatic plant in the Lamiaceae family [1]. Thyme essential oils (EOs) and their derivatives have many uses in foods, pharmaceuticals, and cosmetics due to their antioxidant, antiviral, antifungal, antibacterial, anti-inflammatory, and immunological properties. Thyme’s EOs are natural, safe, and environmentally friendly alternatives to synthetic chemical preservatives and antibiotics since they have no negative effects. Therefore, they are also recognized as natural food additives, chemical preservatives, and feed additives [2] around the world. Thyme EOs can be used as botanical insecticides and have advantages such as efficient herbivore control [3]. Thyme EOs can be transformed into a kind of nanoemulsion, increasing the antibacterial action. The nano-form can be employed as a substitute antimicrobial agent in fish or food products that have been processed or packaged [4]. *T. vulgaris* EO’s effects on orange storage period and penicillium decay were studied and the results indicate that the citrus industry may adopt active packaging to extend the shelf life of oranges for fresh consumption [5]. In conclusion, thyme is widely used in medicines, foods, feed additives, and cosmetics; it is an economic plant with great potential for development.

Thymol, p-cymene, γ-terpinene, carvacrol, geraniol, linalool, 1,8-cineole, (E)-β-caryophyllene, α-terpineol, caryophyllene oxide, borneol, and other terpenoids are the primary components of thyme and oregano essential oils [1,6]. Several thyme species have been reported to exhibit various chemotypes depending on their EOs’ features [1]. Previous studies on the functions of thyme EO compositions mainly focused on thymol. Biosynthetic pathways of thyme monoterpenoids, such as thymol, carvacrol, p-cymene, and γ-terpinene, have been reported [7]. Thyme EOs mainly include thymol (0–86.3%), carvacrol (0–96.2%), p-cymene (0–41.4%), geraniol (0–66.1%), 1,8-cineole (0–67.4%), linalool (0–82.3%), α-terpineol (0–32.0%), and γ-terpinene (0–23.9%) [1,8,9,10,11,12]. In addition to potent antioxidant, antimicrobial, and antifungal properties, thymol, carvacrol, γ-terpinene, and p-cymene can reduce cellular glucose intake and prevent lactate formation [1]. Geraniol is an acyclic monoterpene alcohol in rose [13] that is widely used in perfumes and pharmaceuticals and is a promising gasoline alternative. α-Terpineol and α-terpinyl acetate are mainly used in perfumes, cosmetics, aromatic scents, pharmaceuticals, and food flavoring. In conclusion, different terpene compositions have different functions. To date, extensive analyses of thyme EOs have been conducted and many volatile components have been measured.

Pathogenic bacterial infections, which can be caused by contaminated food and diseased people, are a serious hazard to human health; hence, the need for natural antibacterial agents is urgent. Common pathogenic bacteria, such as *Staphylococcus aureus* and *Escherichia coli*, are found in food-processing firms. These bacteria cause various illnesses and show resistance to traditional treatments [14]. Therefore, it is important to study alternative antibiotics to prevent pathogenic bacterial infection. Plant EOs may be used as an alternative food preservative against foodborne infection. Indeed, aromatic Lamiaceae family plants are widely used as food additives due to their abundance of functional components that prevent degradation and exhibit superior function to standard preservatives [15]. *T. vulgaris* EO showed the highest antimicrobial ability against *S. aureus* and *E. coli* in in vitro antimicrobial tests [16]. The microbial contamination risk in food can be overcome by a synergistic antibacterial effect. Nisin and carvacrol displayed a significantly positive antibacterial synergy, and thus, the required dosage of antibacterials was greatly decreased. Essential oils contain carvacrol, which is a naturally occurring bioactive molecule that also has antioxidant properties. Positively charged nanoparticles enhance the in vitro antimicrobial ability of carvacrol against *S. aureus* and *E. coli*. Compared with a pure chitosan membrane, UiO-66-NH2 containing thymol and carvacrol has a stronger antibacterial function against *S. aureus* and *E. coli* [14]. The development of wild thyme with high thymol and carvacrol contents will lay a good foundation for thyme essential oil functional studies.

Previously, we collected various resources from Chinese native thymes [6] and analyzed thyme’s genome information [7]. In this study, the main goals were to measure the Chinese native thyme EO compositions and determine each ingredient’s content. Through this analysis, we identified many thyme wild germplasms with new EO chemotypes, and thus assessed the chemical profiles of 24 EOs generated from several Chinese native thymes using a multidimensional assessment method that included correlation analysis, cluster analysis, and partial least squares discriminant analysis (PLS-DA). By using DPPH, ABTS, and FRAP studies, the antioxidant properties of the various EO chemotypes were confirmed. Through inhibitory zone diameter, lowest inhibitory concentration, and minimum bactericidal concentration measurements, the antibacterial activities of ten EOs against *S. aureus* and *E. coli* were examined. By using correlation analysis, we also discovered hub chemical compositions associated with the capacity to fight off *S. aureus* and *E. coli*. This study’s findings provide important information for choosing new thyme varieties and some understanding of the functional investigations of new thyme EO chemotypes.

## 2. Results

### 2.1. The Determination of the EO Compositions in Chinese Native Thymes

The EO yields of 24 Chinese native thymes that comprised 10 species *T. quinquecostatus* (Tqu01 and Tqu02), *T. mongolicus* (Tmo01–Tmo05), *T. inaequalis* (Tin01 and Tin02), *T. mandschuricus* (Tma01), *T. curtus* (Tcu01), *T. amurensis* (Tam01), *T. roseus* (Tro01), *T. proximu* (Tpr01), *T. marschallianus* (Tmr01), and *T. altaicus* (Tal01), and two varieties, *T. quinquecostatus* var. *asiaticus* (Tqa01–Tqa04) and *T. quinquecostatus* var. *przewalskii* (Tqp01–Tqp04), were identified (Table 1 and Table 2). The results show that the Chinese native thyme EO yields were obviously different and between 0.3 and 1.6%. Tpr01 showed the highest yield (1.6%) among all the species, and the main components were carvacrol (46.2%), p-cymene (22.2%), γ-terpinene (10.5%), (E)-β-caryophyllene (7.6%), and thymol (4.5%). The lowest oil yield of 0.3% was from Tqa02, and geraniol (58.3%), germacrene D (7.2%), (E)-β-caryophyllene (6.7%), geranyl acetate (5.0%), and bicyclogermacrene (4.3%) were the top five components in this species. The EO yields of Tpr01 (1.6%), Tmo02 (1.4%), Tmo01 (1.3%), Tmo03 (1.3%), and Tqp02 (1.2%) were higher than the other Chinese native thymes; these five Chinese native thymes can be screened as new varieties with high EO yields.

**Figure 1:**
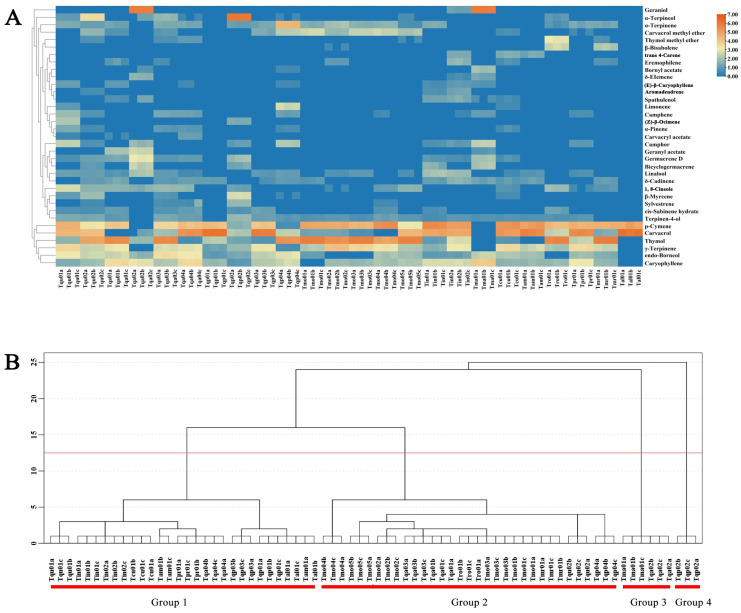
Chinese native thyme essential oil chemical compositions. The compositions are illustrated by a (**A**) heatmap and (**B**) dendrogram.

GC-MS was used to measure the main chemical compositions in the Chinese native thyme EOs. Thirty-five components (relative content > 0.3%) were shown to account for 94.8–100.0% of the total EO compositions, with great differences between species (Table 1 and Table 2). Heatmap analysis was used to evaluate the details of these results. Figure 1A and Table 1 and Table 2 show the main chemical components of the Chinese native thyme EOs, which are defined as more than 5% of the total content. The most abundant EO constituents were terpenes, with a relative percentage > 94.8%, including monoterpenoids and sesquiterpenoids. Monoterpenoids, which contained thymol, carvacrol, geraniol, α-terpineol, and p-cymene, showed the highest percentages in Tmr01 (58.6%), Tqp01 (72.4%), Tma01 (59.5%), Tqp02 (65.4%), and Tin01 (46.0%), whereas sesquiterpenoids, such as (E)-β-caryophyllene and germacrene D, represented the major fractions in Tma01 (14.7%) and Tqa02 (7.2%).

Dendrogram analysis was then performed to classify the Chinese native thyme EOs (Figure 1B). The EO chemical compositions varied greatly between species, where four primary groupings, referred to as groups 1, 2, 3, and 4, were produced. Furthermore, the repeats for each EO sample showed great stability.

### 2.2. Chemodiversity Classification of Chinese Native Thyme EO Chemotypes

**Figure 2:**
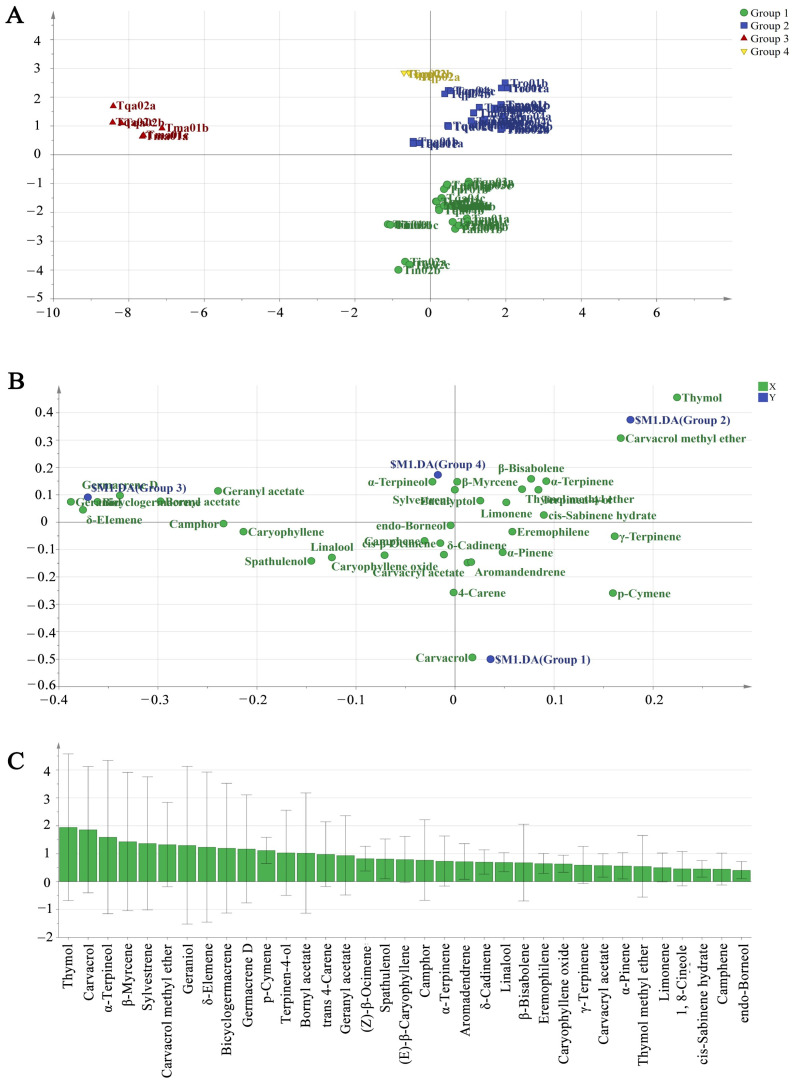
Chemodiversity classification of different Chinese native thyme essential oils. (**A**) Score plot, (**B**) loading plot, and (**C**) VIP values from the PLS-DA analysis based on the four EO groups’ chemical profiles.

The various Chinese native thyme EO compositions were distinguished using a supervised PLS-DA statistical method, as shown by the dendrogram analysis results (Figure 1B and Figure 2). Group 1 comprised 10 species (Tqu01, Tqa04, Tqp01, Tqp03, Tin01, Tin02, Tcu01, Tam01, Tpr01, and Tal01) with a carvacrol-rich type (20.7–72.4%). Moreover, the EOs in group 1 contained abundant p-cymene (15.7–46.0%). Group 2 was a thymol-rich type that comprised Tqu02, Tqa01, Tqa03, Tqp04, Tmo01, Tmo02, Tmo03, Tmo04, Tmo05, Tro01, and Tmr01 (28.9–58.6%). The EOs in group 2 also contained abundant p-cymene (0.6–43.7%). The biosynthetic pathway of monoterpenoids, such as carvacrol, thymol, and p-cymene was analyzed [7]. Group 3 (Tqa02 and Tma01) was characterized as a geraniol-rich (58.3% and 59.5%) type that mainly contained (E)-β-caryophyllene (Tma01, 14.7%) and germacrene D (Tqa02, 7.2%). Group 4 (Tqp02) was characterized as a α-terpineol-rich (65.4%) type.

Chemical markers were screened using the PLS-DA model’s variable importance in the projection (VIP) value, which also served as a parameter for calculating the chemical composition contributions to the PLS-DA model [17]. Figure 2C shows each EO component’s VIP value; vital components were identified based on higher VIP values (≥1). Among these, thymol (1.95), carvacrol (1.86), α-terpineol (1.60), β-myrcene (1.43), sylvestrene (1.37), carvacrol methyl ether (1.33), and geraniol (1.30) stood out as having substantial effects on the PLS-DA model’s classification. Importantly, these substances might be suitable chemical markers for identifying thyme EO chemodiversity

### 2.3. Antioxidant Activity Analysis of Chinese Native Thyme EOs

**Figure 3:**
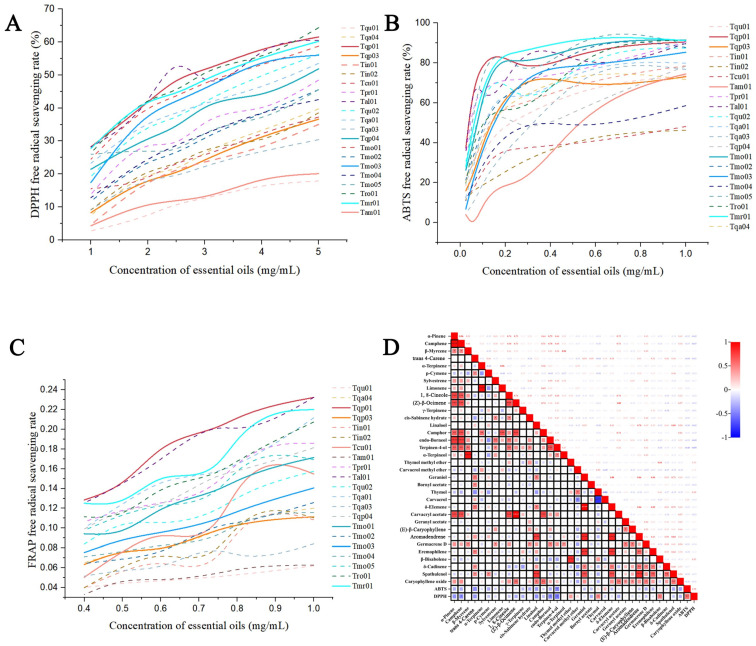
Chinese native thyme essential oils’ antioxidant and correlation analysis results. (**A**) DPPH, (**B**) ABTS, and (**C**) FRAP free-radical-scavenging activities of the essential oils. (**D**) Correlation analysis model based on the composition and ABTS and DPPH antioxidant activities; the number of “*” symbols represents the correlation coefficient. Note: the six representative thymol- and carvacrol-type thyme species are represented by solid lines and the other thyme species are represented by dashed lines.

According to the above results, 24 Chinese native thymes of four chemotypes were selected for antioxidant activity analysis using DPPH, ABTS, and FRAP assays (Figure 3). The results showed that 21 samples had obvious antioxidant activities (all samples except Tqa02, Tma01, and Tqp02), and the EOs’ antioxidant capacities were elevated the concentration of essential oils was increased. The test materials’ ability to scavenge DPPH in the current investigation was evaluated based on their IC50 values, which were defined as the test material concentrations needed to reduce the DPPH solution absorbance at 515 nm to 50% of its initial value. These oils’ IC50 values are listed in Table 3. The DPPH scavenging test revealed that Tqp01, which had a carvacrol concentration of 72.4%, exhibited the strongest antioxidant activity. However, compared with Tqu01, the antioxidant activity was nearly six times more effective at scavenging DPPH (carvacrol content was 20.7%). Tmr01 (thymol content was 58.6%) showed the highest antioxidant activity according to the DPPH, ABTS, and FRAP scavenging tests. This can be attributed to the higher phenolic content of the former than that of the latter. At the same time, the different free-radical-scavenging test results were also different. The ABTS results showed that the antioxidant capacities of the EOs rich in thymol and carvacrol were significantly superior to those rich in geraniol and α-terpineol, and the antioxidant capacities of EOs rich in thymol were a little better than those rich in carvacrol. These results were in agreement with the DPPH test.

The abilities of the thymol-rich- and carvacrol-rich-type EOs to scavenge ABTS free radicals were superior to that of ascorbic acid; although, the DPPH free radical scavenging was less effective than that of ascorbic acid. In comparison with DPPH and ABTS, the FRAP analysis revealed a strong ability to convert Fe^3+^ into Fe^2+^ and substantial antioxidant activity. For Fe^3+^, the reducing abilities of the thymol- and carvacrol-type EOs were stronger than the other chemotypes, and their reducing abilities were also stronger than that of ascorbic acid. The above results reveal that the Chinese native thyme EOs had moderate-to-high antioxidant potentials. In general, the thymol- and carvacrol-type EOs’ antioxidant capacities were obviously better than those of the geraniol- and α-terpineol-type EOs, and even better than that of ascorbic acid in some respects. The correlation analysis showed that the thyme EOs’ antioxidant activities against ABTS and DPPH were positively correlated with thymol and carvacrol, with correlation coefficients of 0.50 and 0.16, respectively (Figure 3D). Furthermore, geraniol and α-terpineol showed obvious negative correlations.

### 2.4. Antibacterial Activity Analysis of the Chinese Native Thyme EOs

**Figure 4:**
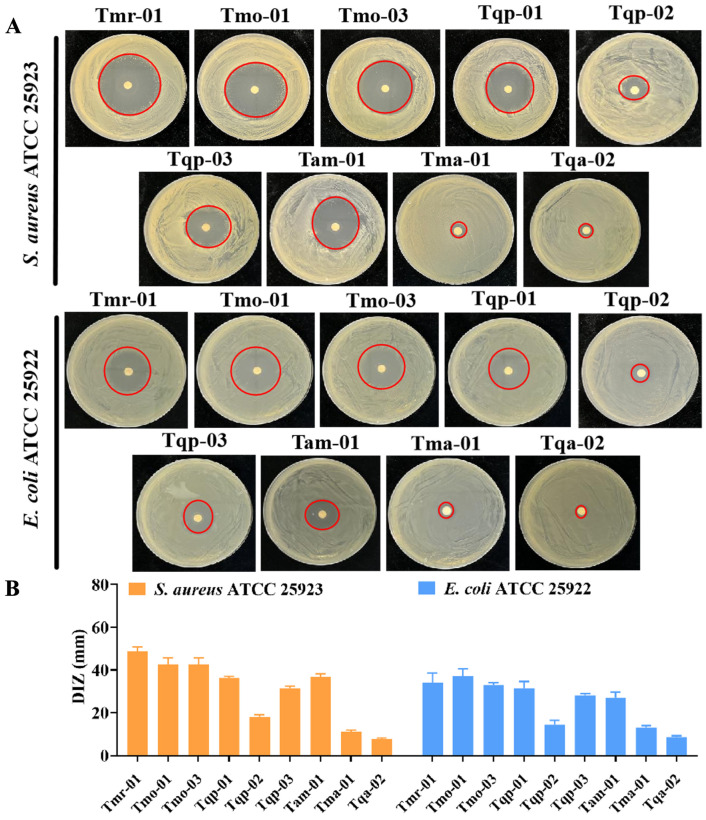
Chinese native thyme essential oils’ antibacterial activities. (**A**) DIZ images and (**B**) statistical data for the essential oils against *S. aureus* and *E. coli*. The discs measured 6 mm in diameter, and the values represent the means ± standard deviations (*p* < 0.05). The red circles represent the degree of antibacterial activity of different essential oils.

DIZs were used to test the various EOs’ antibacterial effects on *S. aureus* ATCC 25923 and *E. coli* ATCC 25922 development (Figure 4). The findings revealed that the EO susceptibility of *S. aureus* and *E. coli* varied, with halos that ranged from 7.67 to 48.67 mm (Figure 4A). The Gram-positive (G+) *S. aureus* continued to be more vulnerable to EOs than the Gram-negative (G−) *E. coli*. (i.e., Tmr01, Tmo01, Tmo03, Tam01, Tqp01, and Tqp03). The initial DIZ analysis results showed that all EOs, except Tma01 and Tqa02, effectively reduced the pathogen development by various degrees. For G− and G+ bacteria, Tmr01, Tmo01, and Tmo03 showed higher antimicrobial activities and larger halos. The DIZs of Tam01, Tqp01, and Tqp03 were very similar and exhibited secondary antibacterial activities. Generally, Tqp02 displayed mild antibacterial activity, while Tma01 and Tqa02 displayed essentially negligible antimicrobial activity. The chemodiversity results led to the conclusion that the thymol-rich Chinese native thyme EOs had a greater antibacterial power than the carvacrol-rich EOs; in contrast, the geraniol- and α-terpineol-rich EOs had lower antibacterial activities. Due to variations in the primary components’ proportions, there also existed variations in the antimicrobial capabilities within a type.

### 2.5. Correlation Analysis

**Figure 5:**
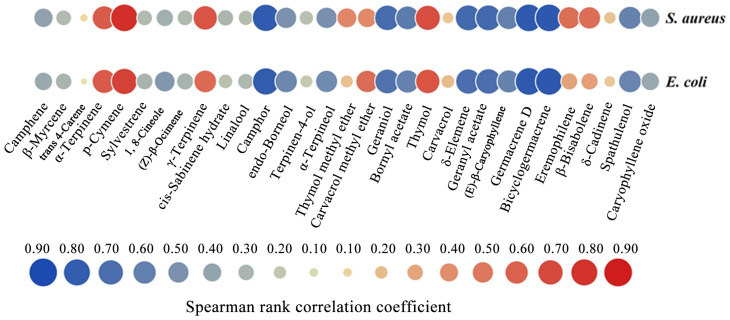
Spearman correlation analysis of Chinese native thyme essential oils’ compositions and antibacterial properties. The correlation strengths are represented by differently colored circles: red circles represent positive correlations, whereas blue circles indicate negative correlations. The circle size represents the correlation strength.

The Spearman rank correlations between the EO chemical compositions and bacteria are represented in Figure 5. P-cymene and thymol, two of the main components found in EOs, showed substantial positive correlations with *S. aureus* and *E. coli*, highlighting their critical functions in antimicrobial action. Interestingly, p-cymene and thymol showed stronger correlations with the G+ bacteria (*S. aureus*, r = 0.82 and 0.68) compared to the G− bacteria (*E. coli*, r = 0.74 and 0.65). This finding suggests that the EO compositions effective against G− and G+ bacteria may differ. Thymol was significantly positively correlated with typical common bacteria. Thus, these findings showing that thymol may be able to prevent harmful microorganisms are intriguing. Additionally, there was a substantial positive correlation between γ-terpinene and α-terpinene with *S. aureus* and *E. coli*. However, we found that germacrene D, bicyclogermacrene, camphor, and geraniol exhibited significantly negative correlations with *S. aureus* and *E. coli*.

## 3. Discussion

Thyme is an important aromatic and medicinal plant with many uses in medicines, foods, feed additives, and cosmetics thanks to their antioxidant, antiviral, antifungal, antibacterial, anti-inflammatory, and immunological properties. In this study, we identified 24 Chinese native thymes’ EO yields and compositions. Among these species, the Tpr01 (1.6%), Tmo02 (1.4%), Tmo01 (1.3%), Tmo03 (1.3%), and Tqp02 (1.2%) EO yields were higher than the other Chinese native thymes. These five Chinese native thymes can be screened as new varieties with high EO yields (Table 1). The EO compositions’ relative contents were different (Figure 1A). For example, the dominant components, i.e., thymol, carvacrol, geraniol, and α-terpineol, had the highest contents in 58.6% (Tmr01), 72.4% (Tqp01), 59.5% (Tma01), and 65.4% (Tqp02), respectively. Notably, EOs are secondary metabolites that are helpful for plant communication and defense and can be influenced by many variables, including pest control, geographical location, environmental factors, harvesting times, fertilization techniques, and extraction techniques [1]. Meanwhile, some genes involved in volatiles production may be impacted over time by internal genetic variables connected to the terpenoids’ biosynthetic pathways. In general, various ecotypes or chemotypes were generated by those factors in the same species [18]. Further research should be undertaken to determine how the internal genetic factors and external environment interact. Thyme EO formation will be well supported by these key terpenoids.

Many of the Chinese native thymes had high thymol and carvacrol contents: Tmo01, Tmo03, Tmo05, Tro01, and Tmr01 had 56.9, 56.1, 54.1, 52.3, and 58.6% thymol contents, respectively, while Tqp01, Tqp03, Tam01, and Tal01 had 72.4, 59.8, 51.3, and 71.7% carvacrol contents, respectively (Table 1). Thymol and carvacrol can suppress lactate formation; lessen cellular glucose uptake; and provide potent antioxidant, antimicrobial, and antifungal effects [1]. The geraniol contents (58.3 and 59.5%, Table 1) were higher than those in rose [13]. *R. damascena* should be further studied as a holy ancient plant with modern uses in perfumery, cuisine, preclinical and clinical investigations, and cosmetics. The geraniol content in 50 rose EOs ranged from 15.8 to 46.6% [19]. The dominant component of Tqp02 was α-terpineol (65.4%), which was higher than that in *Chamaecyparis obtusa* [20]. The geraniol- and α-terpineol-rich EOs from Chinese native thyme germplasms provide a new direction and insight for geraniol functional development and thyme utilization.

Furthermore, we carried out a multidimensional analysis to clarify the Chinese native thyme EOs’ chemical profiles. The PLS-DA statistical method was utilized in the dendrogram analysis to discriminate between the various Chinese native thyme EO compositions, which made it possible to discover the factors that had the greatest influence on the classification of the four groups, as well as the underlying chemical markers. PLS-DA could be used to separate lavender EOs based on quality using 15 compounds that were selected among 170 compounds [21]. Using 50 chemicals from eight different oregano cultivars, PLS-DA could distinguish between different oregano EOs according to quality [17]. The VIP method chose some chemical markers (i.e., carvacrol) that represented the differences in chemical composition between three groups of oregano EOs. In this study, thymol-, carvacrol-, geraniol-, and α-terpineol-rich chemotypes were defined using the main compounds, which were the chemical markers (i.e., thymol, carvacrol, geraniol, and α-terpineol) in the Chinese native thymes. Overall, the composition screening and phytochemical properties assessment of the Chinese native thymes were greatly aided by the EO multidimensional analysis.

DPPH, ABTS, and FRAP antioxidant assays were used to analyze the 24 Chinese native thyme EO samples (Figure 3). Many of the samples showed distinct antioxidant activities, which increased with the increase in the concentration of the essential oils applied. At the same time, the thymol- and carvacrol-rich chemotype EOs’ antioxidant capacities were significantly superior to the other chemotypes. Two monoterpene isomers, i.e., thymol and carvacrol, were isolated from thyme or oregano and displayed anti-inflammatory, antibacterial, antimicrobial, antihypertensive, immunomodulatory, and anticancer activities [22,23]. Additionally, their use in creating functional food has impacted food quality and enhanced human health [18]. Due to their compounds’ high volatility and potent inhibitory effects, their EOs can be employed as an alternative to antimicrobial agents in processed or packaged food. Carvacrol and thymol in products demonstrated their ability to increase the shelf-life of many foods and vegetables [23].

In addition, we found important high-geraniol wild thyme resources in nature (58.3 and 59.5%, Table 1 and Table 2), which laid the foundation for further research on geraniol biosynthesis and function. A valuable monoterpene alcohol called geraniol is used extensively in cosmetics, fragrances, and pharmaceuticals [24,25,26,27,28,29]. Geraniol has the potential to be useful in controlling invasive tumor behavior because it is very successful at generating cytotoxicity and shows AGS migration [30]. Geraniol may prevent renal I/R by inhibiting the TLR2,4/MYD88/NF kappa B pathway, mediating anti-inflammation and Nrf2 pathway activation, and interfering with antioxidative actions [31]. Geraniol has extensive potential as a natural remedy in antibiotic replacement creation and use. The burden of dangerous synthetic larvicides might be reduced by using natural compounds, such as geraniol, which affect oviposition’s chemical ecology [32]. Dietary geraniol supplementation effectively modifies age-related neuroinflammation and oxidative stress in rats, leading to the use of geraniol as a noninvasive natural compound for controlling age- and diet-associated neuronal impairments and toxicity [33]. Furthermore, in combination with vancomycin, geraniol can reduce biofilm adhesion on implants in mice. The potential of geraniol as an anti-MRSA biofilm drug can provide a solution for biofilm infection clinical treatment [34]. In conclusion, geraniol has many medicinal functions, and we will thoroughly study and develop the functions of the geraniol-rich chemotype of thyme EOs.

Thymol- and carvacrol-rich chemotype EOs in 35 chemical compositions of Chinese native thyme EOs were found to have significant antimicrobial activities. Thymol and carvacrol are bioactive monoterpenoids found abundantly in oregano, thyme, and bergamot and have diverse pharmacological benefits. Additionally, most food-borne pathogens are inhibited by these chemical compositions [13]. Some studies focused on the EOs’ antibacterial capacities and mechanisms against the G+ *S. aureus* or G− *E. coli* [35]. Minor EO compositions may have good antibacterial efficiency, according to several studies [36]. Human health is at risk from bacterial food contamination and food poisoning; hence, the need for natural antibacterial agents is urgent. Thymol and carvacrol are both widely regarded as safe and effective antibacterial drugs. The bacterial membrane breakdown, which results in bacterial lysis, intracellular content leaking, and cell death, is the most frequently described antibacterial action mode for both isomers [37]. Zirconium-based metal-organic frameworks (MOFs) were manufactured as loading carriers for thymol and carvacrol and added into chitosan to construct antimicrobial packaging films to develop a premium antimicrobial material [14]. From a resistance perspective, studying EO functions in plants is more valuable than studying individual chemical components because of their potential synergistic effects. Carvacrol administration markedly recovered antioxidant proteins and attenuated kidney histological changes due to fibrosis by targeting oxidative stress and inflammation [38]. Therefore, exploring the new functions of thyme EOs’ main components will be our new research focus.

## 4. Materials and Methods

### 4.1. Plant Materials

Based on previously collected results [6], 24 Chinese native thymes were propagated using the cuttage of 10 species and two varieties (Table 4). Herbarium specimens were authenticated at the Institute of Botany, Chinese Academy of Sciences (IB-CAS). All the Chinese native thymes were cultivated in the experimental field during 2021 at the IB-CAS, Beijing, China.

*S. aureus* ATCC 25923 and *E. coli* ATCC 25922 were acquired from the IB-CAS. These two bacteria strains were kept in Luria Bertani (LB) broth with 25% glycerol (*v*/*v*) at −80 °C. The bacteria strains was cultured in a shake flask with LB broth for 18 h at 37 °C before the antibacterial experiment. 

### 4.2. Essential Oil (EO) Isolation Using Steam Distillation

The aerial plant parts were reaped using cutting propagation in the 2022 full-blossom period and then dried at room temperature. Dried samples were ground to a power. The powdered samples (100 g) were mixed in 1000 mL distilled water and the Chinese native thyme EO was obtained using steam distillation. The Chinese native thyme EO was obtained using steam distillation, which was carried out at 90–100 °C for 90 min. The thyme EO yield (%) was computed as the isolated EO volume (mL) per 100 g of the dry plant material. The thyme EO was dried utilizing anhydrous sodium sulfate before storage at 4 °C [39].

### 4.3. EO Composition Analysis

GC-MS studies of the 24 Chinese native thyme EOs were carried out on an gas chromatograph (Agilent 7890A-7000B, Agilent, USA) fitted with an Agilent 5975C MS detector. The volatiles separation was performed using a capillary column with an HP-5MS core (30 m, 250 μm ID, 0.25 μm film thickness) using the following process: 5 min at 60 °C, followed by 4 °C/min to 220 °C, held for 5 min, and then 60 °C/min to 250 °C, with a temperature of 250 °C for the injector and detector. He was the carrier gas with a flow rate of 1 mL/min, a split ratio of 1:10, an acquisition range of 50–500 m/z in electron-impact mode, an ionization voltage of 70 eV, and a 1 µL sample injection volume. The standardization of the GC peak regions served as the foundation for calculating each compound’s percentage content. The EO compound identification was based on the contrast of the retention indices versus the homologous series of n-alkanes (C7–C40) and mass spectra (MS) from the NIST (v17.0) library [40]. 

### 4.4. Chinese Native Thyme EOs’ Antioxidant Activities

#### 4.4.1. DPPH Free-Radical-Scavenging Activity Assay

As previously reported by [41], the EOs’ radical scavenging effects on 2,2-diphenyl-1-picrylhydrazyl (DPPH) radicals and the EOs’ antioxidant activities were evaluated using the total antioxidant activity DPPH kit (CominBio, Suzhou, China). According to the preliminary experimental results, we found that different EO chemotypes had different antioxidant activities. Therefore, the EOs were diluted with methanol into different concentrations (ascorbic acid as the positive control; 5.0, 4.0, 3.0, 2.5, 2.0, and 1.0 mg/mL). Then, 10 μL of the sample or methanol (the control group) was used to react with 190 μL of the DPPH reagent at room temperature in the dark. We tested the reagent absorbance after the 20 min reaction at 515 nm. The DPPH free-radical-scavenging ratio (%) was calculated as follows: (OD_blank_ − OD_sample_) × 100%/OD_blank_.

#### 4.4.2. ABTS Free-Radical-Scavenging Assay

The total antioxidant activity ABTS kit (CominBio, Suzhou, China) was used to assess the EOs’ antioxidant activities in vitro [42]. According to the preliminary experimental results, the antioxidant activities of different EO chemotypes were different. Therefore, the EOs were diluted with methanol into different concentrations (ascorbic acid as the positive control; 1.0, 0.5, 0.25, 0.1, 0.05, and 0.025 mg/mL). In brief, 190 μL of the reaction reagent was combined with 10 μL of the sample or methanol (the control group) in a 96-well plate. Within 10 min, the absorbance at 734 nm was measured. The blank was determined after mixing 10 μL of methanol with 190 μL of reaction solution. The ABTS free-radical-scavenging ratio (%) was calculated as follows: (OD_blank_ − OD_sample_) × 100%/OD_blank_.

#### 4.4.3. FRAP Reducibility Determination

As previously mentioned, the ferric reducing antioxidant power (FRAP) assay kit (CominBio, Suzhou, China) was used to measure the EO antioxidant activity [12]. The EO was diluted with methanol into various concentrations (ascorbic acid as the positive control; 1.0, 0.8, 0.6, 0.5, 0.4, and 0.2 mg/mL). In the actual processing, 190 μL of the reaction reagent was combined with 10 μL of the sample or methanol (the control group) in a 96-well plate for 20 min. Each sample’s total antioxidant capacity was expressed by comparing the absorbance value at 593 nm with the antioxidant Trolox’s standard curve. The standard fitting equation was y = 0.5327x + 0.0285, R2 = 0.9732, where x is the tocopherol concentration (μmol/mL) and y is ΔOD = ODsample − ODblank.

### 4.5. Chinese Native Thyme EOs’ Antibacterial Activities Against S. aureus and E. coli

The disc diffusion method [43] was used to evaluate the various Chinese natural thyme EOs’ antibacterial effects. Briefly, 100 μL *S. aureus* and *E. coli* suspensions (approximately 107 CFU/mL) were smeared evenly on LB agar plates. Then, we placed the sterilized antimicrobial disks on experiment plates; 10 μL of Chinese native thyme EO was added to the 6 mm disc’s center and then incubated at 37 °C for 24 h. The diameter of the inhibitory zone (DIZ) was measured using vernier calipers (Airaj, Tsingtao, China). Three replicates of each experiment were carried out.

### 4.6. Data Analysis

All data are displayed as the mean ± standard deviation. Variance, hierarchical cluster, and correlation analyses were undertaken using SPSS software (version 25.0; SPSS, Chicago, IL, USA). Heatmap and dendrogram analyses were performed using the R platform. Partial least squares discriminant analysis (PLS-DA) was undertaken using SIMCA software (version 14.1; Umetrics, Umea, Sweden). The half-maximum inhibitory concentration (IC50) of the antioxidant activity was determined using GraphPad Prism (version 7.0; San Diego, CA, USA). Cytoscape (version 3.7.2; National Resource for Network Biology) was used to further visualize the correlation between the DIZ and the thyme EO chemical components using the Spearman rank correlation. When the *p*-values were less than 0.05, the experimental results were deemed statistically significant.

## 5. Conclusions

Antioxidant and antibacterial activities are present in thyme EOs and their mixtures. This study evaluated the chemical compositions, chemotypes, antioxidant, and antibacterial activities of various Chinese native thyme EOs. The findings revealed that the EOs exhibited significant chemodiversity, and four main groups were distinguished: carvacrol-, thymol-, geraniol-, and α-terpineol-rich. The results show that the thymol- and carvacrol-rich EOs’ antioxidant capacities were significantly superior to those of the geraniol- and α-terpineol-rich EOs. Additionally, the thymol- and carvacrol-rich EO groups displayed strong inhibitory effects on common food-borne pathogenic microorganism development. The potential antibacterial activities with main chemical compositions and their synergistic effects were revealed using a correlation network analysis. Overall, our findings may help develop a mechanism for evaluating EOs’ chemical profiles and antioxidant and antibacterial activities, and it has laid a strong foundation for the breeding of high-quality thyme with more advantageous commercial properties for usage in the food industry. Although this study revealed that EOs have potential as antibiotic alternatives, their antibacterial applications are constrained by their powerful flavor, volatility, and chemical instability. Therefore, embedding thyme EOs in nanomaterials can reduce EOs’ volatilization and allow for broad application prospects.

## Figures and Tables

**Table 1:** Relative volatile terpenoid concentrations in 12 Chinese native thymes’ (*T. quinquecostatus*, *T. quinquecostatus* var. *asiaticus*, *T. quinquecostatus* var. *przewalskii*, and *T. inaequalis*) essential oils.

Terpenoid	Empirical Formula	RT	RI	Concentration (%)											
				Tqu01	Tqu02	Tqa01	Tqa02	Tqa03	Tqa04	Tqp01	Tqp02	Tqp03	Tqp04	Tin01	Tin02
α-Pinene	C_10_H_16_	7.03	929	1.6 ± 0.0	0.5 ± 0.0	-	-	0.9 ± 0.1	0.8 ± 0.1	-	-	-	0.5 ± 0.1	-	-
Camphene	C_10_H_16_	7.56	952	2.6 ± 0.0	0.7 ± 0.0	-	-	1.4 ± 0.1	1.2 ± 0.1	-	-	-	1.2 ± 0.1	-	0.4 ± 0.0
β-Myrcene	C_10_H_16_	9.16	991	1.0 ± 0.0	2.7 ± 0.0	-	-	0.7 ± 0.1	-	-	5.8 ± 0.2	-	0.4 ± 0.0	-	-
*trans* 4-Carene	C_10_H_16_	10.06	1009	-	-	-	-	-	-	-	-	-	-	-	1.6 ± 0.0
α-Terpinene	C_10_H_16_	10.06	1017	1.7 ± 0.0	1.1 ± 0.0	1.3 ± 0.2	-	1.6 ± 0.1	1.5 ± 0.1	-	1.1 ± 0.1	1.4 ± 0.2	21.1 ± 0.7	1.7 ± 0.1	-
* p-Cymene	C_10_H_14_	10.38	1021	23.0 ± 0.1	8.7 ± 0.4	15.6 ± 0.9	-	11.6 ± 0.1	17.3 ± 1.5	18.0 ± 1.4	2.0 ± 0.2	15.7 ± 1.0	0.6 ± 0.0	46.0 ± 1.5	33.2 ± 1.1
Sylvestrene	C_10_H_16_	10.53	1027	-	0.5 ± 0.0	-	-	0.6 ± 0.0	1.0 ± 0.9	-	2.5 ± 0.1	-	-	-	-
1,8-Cineole	C_10_H_18_O	10.62	1032	6.7 ± 0.0	2.1 ± 0.0	2.1 ± 0.0	1.8 ± 0.3	2.5 ± 0.1	2.3 ± 0.1	-	1.2 ± 0.1	-	5.3 ± 0.6	-	-
(Z)-β-Ocimene	C_10_H_16_	11.23	1038	3.4 ± 0.0	-	-	-	-	-	-	1.6 ± 0.1	-	-	-	-
* γ-Terpinene	C_10_H_16_	11.72	1060	11.5 ± 0.1	4.8 ± 0.1	7.5 ± 0.5	-	5.8 ± 0.3	6.7 ± 0.4	1.2 ± 0.1	2.5 ± 0.0	8.8 ± 0.1	0.5 ± 0.0	2.0 ± 0.2	7.7 ± 0.6
*cis*-Sabinene hydrate	C_10_H_18_O	12.09	1070	0.6 ± 0.0	0.6 ± 0.0	1.0 ± 0.1		0.7 ± 0.0	1.1 ± 0.1		0.7 ± 0.0	1.2 ± 0.1	-	0.6 ± 0.1	-
* Linalool	C_10_H_18_O	13.32	1099	1.2 ± 0.0	1.1 ± 0.1	0.7 ± 0.1	3.3 ± 0.3	0.9 ± 0.0	0.5 ± 0.1	-	-	0.86 ± 0.1	1.0 ± 0.1	3.2 ± 0.3	2.6 ± 0.1
* Camphor	C_10_H_16_O	15.06	1143	4.4 ± 0.0	0.7 ± 0.0		2.3 ± 0.3				1.1 ± 0.1		3.6 ± 0.3	0.7 ± 0.1	1.0 ± 0.1
endo-Borneol	C_10_H_18_O	15.80	1167	7.9 ± 0.1	5.1 ± 0.4	5.1 ± 0.7	2.2 ± 0.4	1.0 ± 0.3	7.9 ± 0.2	0.9 ± 0.1	1.5 ± 0.0	4.4 ± 0.3	6.5 ± 0.6	0.9 ± 0.1	1.2 ± 0.2
Terpinen-4-ol	C_10_H_18_O	16.31	1177	1.6 ± 0.0	1.5 ± 0.1	0.9 ± 0.1	-	1.5 ± 0.0	1.1 ± 0.0	-	2.5 ± 0.1	1.2 ± 0.0	0.9 ± 0.1	0.4 ± 0.0	0.8 ± 0.0
* α-Terpineol	C_10_H_18_O	16.77	1189	1.0 ± 0.0	10.5 ± 0.1	-	1.2 ± 0.0	2.4 ± 0.1	0.4 ± 0.0	-	65.4 ± 1.0	-	0.4 ± 0.0	-	-
Thymol methyl ether	C_11_H_16_O	18.44	1235	-	0.6 ± 0.0	-	-	0.6 ± 0.0	1.0 ± 0.0	-	-	-	-	-	-
Carvacrol methyl ether	C_11_H_16_O	18.77	1244	-	2.3 ± 0.0	0.7 ± 0.0	-	0.9 ± 0.1	-	-	-	1.6 ± 0.2	4.0 ± 0.0	-	-
* Geraniol	C_10_H_18_O	19.19	1255	-	-	-	58.3 ± 2.0	-	-	-	-	-	-	-	1.2 ± 0.1
Bornyl acetate	C_12_H_20_O_2_	20.18	1287	-	-	-	0.7 ± 0.0	-	-	-	-	-	0.5 ± 0.0	-	-
* Thymol	C_10_H_14_O	20.38	1291	2.1 ± 0.0	28.9 ± 0.5	44.5 ± 2.2	2.5 ± 0.1	48.4 ± 1.4	0.5 ± 0.0	3.9 ± 0.3	1.3 ± 0.1	0.7 ± 0.0	40.2 ± 1.2	1.0 ± 0.0	5.9 ± 0.3
* Carvacrol	C_10_H_14_O	20.70	1299	20.7 ± 0.1	17.6 ± 0.4	-	1.3 ± 0.1	2.9 ± 0.2	41.0 ± 2.7	72.4 ± 1.0	3.5 ± 0.3	59.8 ± 1.7	4.3 ± 0.1	28.8 ± 1.7	26.9 ± 0.8
δ-EIemene	C_15_H_24_	22.01	1338	-	-	-	2.0 ± 0.2	-	-	-	-	-	-	-	0.5 ± 0.1
Carvacryl acetate	C_12_H_16_O_2_	22.95	1336	0.4 ± 0.0	-	-	-	-	-	-	-	-	-	-	-
Geranyl acetate	C_12_H_20_O_2_	23.48	1385	-	-	3.2 ± 0.1	5.0 ± 0.3	-	-	-	-	-	-	-	-
* (E)-β-Caryophyllene	C_15_H_24_	24.64	1419	1.7 ± 0.0	2.8 ± 0.2	10.5 ± 0.8	6.7 ± 0.5	3.5 ± 0.2	11.1 ± 0.3	2.5 ± 0.2	0.9 ± 0.0	3.7 ± 0.1	5.4 ± 0.7	5.7 ± 0.1	5.5 ± 0.5
Aromadendrene	C_15_H_24_	25.33	1440	-	0.5 ± 0.0	-	-	-	-	-	-	-	-	0.6 ± 0.0	1.4 ± 0.1
Germacrene D	C_15_H_24_	26.57	1481	0.6 ± 0.0	1.2 ± 0.0	1.5 ± 0.1	7.2 ± 0.4	0.8 ± 0.0	1.0 ± 0.0	-	2.8 ± 0.4	-	-	0.8 ± 0.1	0.6 ± 0.1
Bicyclogermacrene	C_15_H_24_	27.04	1499	-	1.0 ± 0.1	-	4.3 ± 0.7	-	-	-	1.5 ± 0.2	-	-	1.9 ± 0.5	-
Eremophilene	C_15_H_24_	27.09	1499	-	-	0.9 ± 0.4	-	0.6 ± 0.0	-	-	-	-	-	-	2.2 ± 0.3
β-Bisabolene	C_15_H_24_	27.39	1509	-	-	-	-	-	-	-	-	-	-	-	-
δ-Cadinene	C_15_H_26_	27.94	1524	-	1.0 ± 0.1	1.4 ± 0.2	-	0.9 ± 0.0	1.1 ± 0.0	1.1 ± 0.1	-	0.6 ± 0.0	0.9 ± 0.1	1.1 ± 0.1	2.0 ± 0.2
Spathulenol	C_15_H_24_O	29.45	1580	-	-	0.6 ± 0.0	1.5 ± 0.0	-	-	-	-	-	-	1.4 ± 0.0	1.6 ± 0.3
Caryophyllene oxide	C_15_H_24_O	29.62	1581	1.1 ± 0.0	-	0.9 ± 0.0	-	-	0.4 ± 0.0	-	-	-	0.4 ± 0.0	0.5 ± 0.1	0.9 ± 0.0
Total				94.8 ± 0.0	96.5 ± 0.1	98.7 ± 0.1	98.9 ± 1.0	98.8 ± 0.5	97.9 ± 0.6	100.0 ± 0.0	97.8 ± 0.5	99.6 ± 0.4	97.4 ± 0.2	97.4 ± 0.2	97.1 ± 0.3
EO yields (%)				0.5	0.7	1.0	0.3	0.8	0.9	0.7	1.2	0.8	0.7	0.8	0.6

Notes: RT, retention time; RI, retention index; values are reported as the mean ± standard deviation of three parallel experiments; and “-” means a composition relative content < 1.0%. Compound identification was based on the NIST 17 mass spectral database and RI values; * components were identified using authentic standard components.

**Table 2:** Relative volatile terpenoid concentrations in 12 Chinese native thymes’ (*T. mongolicus*, *T. mandschuricus*, *T. curtus*, *T. amurensis*, *T. roseus*, *T. proximu*, *T. marschallianus*, and *T. altaicus*) essential oils.

Terpenoid	Empirical Formula	RT	RI	Concentration (%)											
				Tmo01	Tmo02	Tmo03	Tmo04	Tmo05	Tma01	Tcu01	Tam01	Tro01	Tpr01	Tmr01	Tal01
α-Pinene	C_10_H_16_	7.03	929	-	-	-	-	-	-	0.6 ± 0.2	-	-	-	-	-
Camphene	C_10_H_16_	7.56	952	-	-	-	0.5 ± 0.1	-	1.1 ± 0.1	1.1 ± 0.1	-	-	0.6 ± 0.0	-	-
β-Myrcene	C_10_H_16_	9.16	991	-	-	-	-	-	-	0.5 ± 0.0	-	-	-	-	-
*trans* 4-Carene	C_10_H_16_	10.06	1009	-	-	-	-	-	-	1.7 ± 0.2	1.5 ± 0.3	-	-	-	-
α-Terpinene	C_10_H_16_	10.06	1017	1.8 ± 0.2	3.1 ± 0.3	1.9 ± 0.1	2.2 ± 0.1	0.6 ± 0.0	-	-	-	-	1.5 ± 0.3	1.5 ± 0.1	-
* p-Cymene	C_10_H_14_	10.38	1021	27.9 ± 0.7	26.6 ± 1.0	21.0 ± 0.9	43.7 ± 2.3	7.6 ± 0.3	-	38.7 ± 2.5	33.7 ± 5.1	19.2 ± 1.9	22.2 ± 1.5	22.8 ± 0.9	28.0 ± 4.8
Sylvestrene	C_10_H_16_	10.53	1027	-	-	-	0.45 ± 0.01	-	-	-	-	-	-	-	-
1,8-Cineole	C_10_H_18_O	10.62	1032	-	0.6 ± 0.0	-	1.0 ± 0.0	2.2 ± 0.1	-	1.2 ± 0.1	-	2.6 ± 0.2	0.5 ± 0.0	0.8 ± 0.1	-
(Z)-β-Ocimene	C_10_H_16_	11.23	1038	-	-	-	-	-	-	-		-	-	-	-
* γ-Terpinene	C_10_H_16_	11.72	1060	3.7 ± 0.3	17.6 ± 0.7	11.5 ± 0.5	8.0 ± 1.2	3.5 ± 0.1	-	10.8 ± 0.6	5.0 ± 0.9	3.1 ± 0.3	10.5 ± 0.7	4.8 ± 0.5	-
*cis*-Sabinene hydrate	C_10_H_18_O	12.09	1070	-	-	-	0.7 ± 0.1	-	-	-	-	1.2 ± 0.2	-	-	
* Linalool	C_10_H_18_O	13.32	1099	-	0.7 ± 0.0	0.9 ± 0.0	-	0.4 ± 0.0	-	0.3 ± 0.0	0.8 ± 0.0	-	0.6 ± 0.1	-	-
* Camphor	C_10_H_16_O	15.06	1143				-	-	4.4 ± 0.2				0.9 ± 0.0		
endo-Borneol	C_10_H_18_O	15.80	1167	1.1 ± 0.0	-	-	3.2 ± 0.4	2.7 ± 0.2	4.7 ± 0.5	3.4 ± 0.2	2.9 ± 0.1	-	2.8 ± 0.2	1.8 ± 0.2	-
Terpinen-4-ol	C_10_H_18_O	16.31	1177	0.7 ± 0.1	0.9 ± 0.0	0.6 ± 0.0	1.2 ± 0.1	0.8 ± 0.0	0.7 ± 0.1	0.7 ± 0.1	0.7 ± 0.1	0.7 ± 0.1	0.8 ± 0.1	-	-
* α-Terpineol	C_10_H_18_O	16.77	1189	-	-	-	-	0.6 ± 0.0	-	-	-	0.9 ± 0.1	-	-	-
Thymol methyl ether	C_11_H_16_O	18.44	1235	-	-	-	-	0.6 ± 0.1	-	-	-	7.0 ± 0.6	-	0.7 ± 0.1	-
Carvacrol methyl ether	C_11_H_16_O	18.77	1244	5.3 ± 0.4	2.5 ± 0.1	4.5 ± 0.1	4.8 ± 0.4	3.3 ± 0.3	-	0.6 ± 0.0	-	-	0.5 ± 0.0	-	-
* Geraniol	C_10_H_18_O	19.19	1255	-	-	-	-	-	59.5 ± 2.0	-	-	-	-	-	-
Bornyl acetate	C_12_H_20_O_2_	20.18	1287	-	-	-	-	-	-	-	2.9 ± 0.3	-	-	-	-
* Thymol	C_10_H_14_O	20.38	1291	56.9 ± 0.1	41.9 ± 1.7	56.1 ± 1.1	30.4 ± 2.0	54.1 ± 1.2	-	0.8 ± 0.0	-	52.3 ± 2.6	4.5 ± 0.4	58.6 ± 0.8	-
* Carvacrol	C_10_H_14_O	20.70	1299	1.3 ± 0.1	1.4 ± 0.1	0.6 ± 0.0	-	20.2 ± 0.7	-	34.8 ± 3.6	51.3 ± 6.8	4.6 ± 0.5	46.2 ± 2.3	1.7 ± 0.2	71.7 ± 4.8
δ-EIemene	C_15_H_24_	22.01	1338	-	-	-	-	-	1.4 ± 0.1	-	-	-	-	-	-
Carvacryl acetate	C_12_H_16_O_2_	22.95	1336	-	-	-	-	-	-	-	-	-	-	-	-
Geranyl acetate	C_12_H_20_O_2_	23.48	1385	-	-	-	-	-	0.8 ± 0.1	-	-	-	-	-	-
* (E)-β-Caryophyllene	C_15_H_24_	24.64	1419	-	2.7 ± 0.1	2.4 ± 0.2	3.1 ± 0.4	3.4 ± 0.2	14.7 ± 1.0	2.2 ± 0.2	2.4 ± 0.3	2.5 ± 0.2	7.6 ± 0.3	2.3 ± 0.2	-
Aromadendrene	C_15_H_24_	25.33	1440		-	-	-	-	-	-	-		-	-	-
Germacrene D	C_15_H_24_	26.57	1481	-	-	-	-	-	3.1 ± 0.2	0.7 ± 0.2	-	-	-	-	-
Bicyclogermacrene	C_15_H_24_	27.04	1499	-	-	-	-	-	3.4 ± 0.4	-	-	-	-	-	-
Eremophilene	C_15_H_24_	27.09	1499	-	0.9 ± 0.0	-	-	-	-	0.6 ± 0.1	-	-	-	0.7 ± 0.1	-
β-Bisabolene	C_15_H_24_	27.39	1509	-	0.5 ± 0.0	-	-	-	-	-	-	5.0 ± 0.7	-	3.5 ± 0.4	-
δ-Cadinene	C_15_H_26_	27.94	1524	0.8 ± 0.1	-	-	-	-	1.3 ± 0.2	0.9 ± 0.2	1.1 ± 0.2	-	-	1.1 ± 0.1	-
Spathulenol	C_15_H_24_O	29.45	1580	-	-	-	-	-	0.7 ± 0.0	0.5 ± 0.1	-	-	-	-	-
Caryophyllene oxide	C_15_H_24_O	29.62	1581	-	-	-	-	-	0.7 ± 0.0	-	-	-	-	-	-
Total				99.6 ± 0.4	98.7 ± 0.2	99.6 ± 0.3	99.0 ± 0.6	99.6 ± 0.7	99.0 ± 0.3	99.0 ± 0.1	99.3 ± 0.6	99.8 ± 0.3	98.9 ± 0.0	100.0 ± 0.0	100.0 ± 0.0
EO yields (%)				1.3	1.4	1.3	0.8	0.9	1.1	1.1	1.0	1.1	1.6	0.7	1.0

Notes: RT, retention time; RI, retention index; values are reported as mean ± standard deviation of three parallel experiments; and“-” means a composition relative content < 1.0%. Compound identification was based on the NIST 17 mass spectral database and RI values; * components were identified using authentic standard components.

**Table 3:** Chinese native thyme essential oils’ radical-scavenging activities.

Sample ID	Radical-Scavenging Activity	
	DPPH/IC50 (mg/mL)	ABTS/IC50 (mg/mL)
Tqu01	19.35	0.24
Tqu02	4.01	0.08
Tqa01	4.47	0.15
Tqa02	-	-
Tqa03	13.06	0.32
Tqa04	7.06	0.17
Tqp01	2.83	0.04
Tqp02	-	-
Tqp03	8.12	0.17
Tqp04	4.92	0.22
Tmo01	3.36	0.07
Tmo02	5.99	0.11
Tmo03	3.58	0.16
Tmo04	6.59	0.48
Tmo05	9.32	0.05
Tin01	8.41	0.16
Tin02	8.12	1.23
Tma01	-	-
Tcu01	10.04	1.06
Tam01	19.95	0.54
Tro01	2.91	0.12
Tpr01	5.51	0.05
Tmr01	3.12	0.06
Tal01	-	-

**Table 4:** Ecological factors for Chinese native thymes.

Sample ID	Species	Location	Collection Site	Altitude (m)	Longitude (E)	Latitude (N)	Slope	Soil Texture	Habitat Type
Tqu01	*Thymus quinquecostatus*	Hebei Province	Jinhekou Village, Yu County	1046.00	114°55′3″	39°57′1″	Sunny floodplain	Sandy loam	Floodplain in front of a mountain with gravelly sandy land; the main companion plants were *Allium polyrhizum*, *Cymbaria dahurica*, and *Ephedra intermedia*
Tqu02	*Thymus quinquecostatus*	Inner Mongolia Autonomous Region	Zhenglan Banner	1415.66	116°9′20″	42°12′11″	Sunny slope	Sandy loam	The main companion plants were *Soutellari baiclensis*, *Iris locyzii*, *Rhaponticum unifloru*, and *Alliium* sp.
Tqa01	*Thymus quinquecostatus* var. *asiaticus*	Jilin Province	Songyuan City	170.00	124°1′52″	44°36′52″	Sunny slope	Sandy loam	Sandy land formed by deciduous grass; the main companion plants were *Potentilla anserina* and *Gueldenstaedtia verna* subsp. *multiflora*
Tqa02	*Thymus quinquecostatus* var. *asiaticus*	Inner Mongolia Autonomous Region	Zhenglan Banner	1373.00	116°1′43″	42°22′49″	Roadside slope	Sandy loam	Roadside grassland with sandy loam rich in humus
Tqa03	*Thymus quinquecostatus* var. *asiaticus*	Inner Mongolia Autonomous Region	Wuhe Erqin Aobao Forest Farm, Zhenglan Banner	1427.05	116°9′44″	42°30′19″	Roadside gentle slope	Dry sandy loam	Dry sandy land formed after grassland degradation
Tqa04	*Thymus quinquecostatus* var. *asiaticus*	Shanxi Province	Zuoyun County, Datong City	1319.00	112°43′43″	40°6′33″	Sunny slope	Sandy loam	The main companion plants were *Hippophae rhamnoides* and *Lespedeza davurica*
Tqp01	*Thymus quinquecostatus* var. *przewalskii*	Hebei Province	Small Wutai Jinhekou Scenic Spot, Yu County	1117.00	114°33′55″	39°33′46″	Half-sunny slope	Sandy loam	Between roadside rock walls or rock crevices; the main companion plants were *Selaginella sinensis*, *Selaginella sanguinolenta*, and *Spiraea pubescens*
Tqp02	*Thymus quinquecostatus* var. *przewalskii*	Inner Mongolia Autonomous Region	Duolun Reservoir, Duolun County	1269.13	116°38′44″	42°11′44″	Sunny slope	Sandy loam	The main companion plants were *Allium senescens*, *Patrinia rupestris* subsp. *scabra*, and *Spiraea pubescens*
Tqp03	*Thymus quinquecostatus* var. *przewalskii*	Shanxi Province	Fucheng Town, Linchuan County, Jincheng City	1093.00	113°7′42″	36°39′53″	Sunny steep slope	Calcareous sandy loam	Rocky beach by a cliff
Tqp04	*Thymus quinquecostatus* var. *przewalskii*	Shanxi Province	Xiangshan Temple, Hequ County	926.57	111°13′33″	39°24′24″	Sunny slope	Sandy loam	A gravelly yellow sand formed by stratified rock weathering
Tmo01	*Thymus mongolicus*	Beijing Municipal	Baihua Mountain, Fangshan District	1891.48	115°36′34″	39°51′10″	Roadside sunny slope	Sandy loam	Gravel and stone crevices growth, and a local red ant formation of the associated mound
Tmo02	*Thymus mongolicus*	Beijing Municipal	Baihua Mountain, Fangshan District	1935.03	115°35′57″	39°50′10″	Half-sunny slope	Sandy loam	Baicao bank southeast side; found in a crack in the rocks by the roadside
Tmo03	*Thymus mongolicus*	Beijing Municipal	Baihua Mountain, Fangshan District	1809.23	115°35′42″	39°49′21″	Sunny slope	Sandy loam	Roadside rock crevice at the southwest edge of Baicao bank; it is often associated with mounds formed by a local species of red ant
Tmo04	*Thymus mongolicus*	Beijing Municipal	Dongling Mountain, Mentougou District	1915.00	115°28′29″	40°19′31″	Sunny slope	Sandy loam	The sun was leeward with many rocks, well-drained hillsides, or rock joints; sandy loam rich in humus
Tmo05	*Thymus mongolicus*	Ningxia Hui Autonomous Region	Jingyuan County, Guyuan City	2560.00	106°12′41″	35°29′43″	Sunny slope	Sandy loam	Gravel sandy ground formed by weathering sandstone by the highway; the main companion plant was *Stachys sieboldii*
Tin01	*Thymus inaequalis*	Heilongjiang Province	Huma County	440.00	124°1′8″	52°14′15″	Dry sunny slope	Sandy loam	The main companion plants were *Orostachys cartilaginea*, *Thymus inaequalis*, and *Thymus amurensis*
Tin02	*Thymus inaequalis*	Heilongjiang Province	Forest botanical garden	150.00	126°16′	45°45′	Roadside slope	Sandy loam	Roadside slope in the botanical garden area
Tma01	*Thymus mandschuricus*	Heilongjiang Province	Maoer Mountain, Shangzhi City	800.00	127°32′3″	45°20′3″	Half-sunny slope	Loam	Rocky cracks on top of volcanic rock with medium-acid soil with gravel
Tcu01	*Thymus curtus*	Heilongjiang Province	Huma County	440.00	124°1′8″	52°14′15″	Dry sunny slope	Sandy loam	The main companion plants were *Orostachys cartilaginea*, *Thymus inaequalis,* and *Thymus amurensis*
Tam01	*Thymus amurensis*	Heilongjiang Province	Huma County	440.00	124°1′8″	52°14′15″	Dry sunny slope	Sandy loam	The main companion plants were *Orostachys cartilaginea*, *Thymus curtus,* and *Thymus amurensis*
Tro01	*Thymus roseus*	Xinjiang Uygur Autonomous Region	Sailimu Lake, Yining City	2038.09	81°16′9″	44°39′42″	Slope	Sandy loam	On well-drained sandy loam slopes along the shore of the lake; the main companion plants were *Cares* sp. and *Allium polyrhizum*
Tpr01	*Thymus proximus*	Xinjiang Uygur Autonomous Region	Awuzan Ditch, Yining County	>1637.96	81°43′35″	44°8′57″	Sunny slope	Loam	On rock walls or between rock crevices above the snow-ridge spruce line, rich in humus loam; the main companion plants were *Eremurus chinensis*, *Ephedra equisetina*, and *Hylotelephium ewersii*
Tmr01	*Thymus marschallianus*	Xinjiang Uygur Autonomous Region	Tuolasu Grassland, Yining County	1792.00	81°43′52″	44°15′58″	Grassy slope	Gravelly yellow sand	On an alpine grassland open slope; the main companion plants were *Artemisia* sp., *Stipa* sp., *Aneurolepidium* sp., and *Dracocephalum* sp.
Tal01	*Thymus altaicus*	Xinjiang Uygur Autonomous Region	Awuzan Ditch, Yining County	>1637.96	81°43′35″	44°8′57″	Sunny slope	Loam	On rock walls or between rock crevices above the lowest snow-ridge spruce line, rich in humus loam; the main companion plants were *Eremurus chinensis*, *Ephedra equisetina*, and *Hylotelephium ewersii*

## Data Availability

The data presented in this study are available on request from the corresponding authors.
